# Adnexal mass: diagnosis and management: Number 1 - July 2020

**DOI:** 10.1055/s-0040-1715547

**Published:** 2020-07

**Authors:** Jesus Paula Carvalho, Renato Moretti-Marques, Agnaldo Lopes da Silva Filho

**Affiliations:** Faculdade de Medicina, Universidade de São Paulo, São Paulo, SP, Brazil.; Hospital Israelita Albert Einstein, São Paulo, SP, Brazil.; Faculdade de Medicina, Universidade Federal de Minas Gerais, Belo Horizonte, MG, Brazil.

## Key points

- Adnexal masses occurred in women of all age groups, and their etiology and frequency vary age accordingly.
- Most of the adnexal masses are benign, without symptoms diagnosed incidentally, and can have expectant management.
- Otherwise, ovarian cancer is an adnexal mass with poor prognosis and must be managed quickly in an appropriate setting.
- Correct differential diagnoses of benign and malignant mass matter.
- Panels of biomarkers is not sufficient for the initial evaluation of an adnexal mass.
- Transvaginal ultrasonography is the single most effective way of evaluating an ovarian mass.
- Ovarian cancer patients referred to a cancer center for further Management experience the best outcomes.

## Recommendations

- Transvaginal ultrasonography is the single most effective way of evaluating an ovarian mass. Computed tomography (CT), magnetic resonance imaging (MRI), and positron emission tomography (PET) not recommended in the initial evaluation of adnexal masses.
- The suspicious ovarian cysts should be initially assessed by measuring serum CA125 level and transvaginal ultrasound scan.
- Spillage of cyst contents should be avoided preoperative and intraoperatively. Assessment cannot preclude malignancy.
- Frozen sections for the intraoperative diagnosis of a suspicious adnexal mass could be useful when availability and patient preference allow.
- Malignancy histology revealed during or after diagnostic laparoscopy; the comprehensive surgical medical report belongs to the patient, and images should move to a cancer center for further management.
- Consider opportunistic salpingectomy as risk reduced surgery for ovarian cancer during benign operation.

## Background

Tumoral masses originating from the ovaries, fallopian tubes, and structures around these organs are called adnexal masses, which occur in women of all ages, and their etiology and frequency range age accordingly. The adnexal mass may come from functional or physiological changes, inflammatory processes, endometriosis, benign and malignant tumor. Moreover, the differential diagnosis from a non-gynecologic disorder has to be done.^1^ The actual incidence of adnexal masses in the general population is unknown since most of these are asymptomatic and undiagnosed. Usually are detected on physical examination or pelvic imaging screening. Less commonly, an adnexal mass may present with symptoms of acute or intermittent pain.^1^

The incidence and mortality due to ovarian cancer have remained stable over the past three decades and represent the leading cause of death from malignant neoplasm of the female genital tract in developed countries.^2^ The literature does not support routine screening for ovarian cancer in the general population, and any professional society does not currently recommend it.^3^

The diagnosis of adnexal mass in women with pelvic symptoms or incidentally represents a routine in gynecological practice and often presents diagnostic and management dilemmas.^1^ The mainstream to management of adnexal masses is excluding malignancies. The characterization of malignancy findings on the image (TVUS or MRI) is the key since women with ovarian cancer should preferably be treated in oncological referral centers as soon as possible. The false-negative rates are uncommon and benign adnexal masses can have expectant management or undergo conservative surgery in general hospitals.^4^

## How to differentiate benign from malignant disease?

Estimate the malignancy risk index is essential to assess an adnexal mass. The definition based on image characteristics, in addition to age, oncologic personal and family history, symptoms, findings on physical examination, and levels of tumor markers.^2^ Thus, patients are classified as high or low risk for malignancies (Chart 1). Specific attention should be given to risk or protective factors for ovarian malignancy revealed on medical history symptoms suggestive of ovarian malignancy, and a family history of ovarian, bowel or breast cancer.^5^ ^6^ The complete physical examination, including performance status, body mass index, palpable peripheric lymph nodes, and leg lymphedema evaluation, are useful to characterize the patient. The clinical scan of the abdomen brings the most interpretive signs to malignancy suspicion as ascites, abdominopelvic palpable mass, mobility, combined to its anatomic relations with the uterus, bladder, rectum-sigmoid evaluated by vaginal examination.^5^ Imaging and laboratory testing may clarify the suspected etiology of a pelvic mass. Pregnancy testing obtained in reproductive-aged women is mandatory.^1^

**Chart 1:** Risk stratification of adnexal masses

Characteristic	High-risk	Low-risk
Age	> 50 years	<50 years
Family history	Present	Absent
Symptoms	Persistent and multiple	Absent
Physical examination findings	Large, fixed, irregular mass, evidence of ascites or metastases	Not suggestive of high risk
Tumor markers	Elevated	Normal
Ultrasound findings	≥10 cm, thick, multilocular septation, increased and / or mixed echogenicity and / or solid component, papillary growths present	<10 cm, absent or fine septum (1–2 mm), unilocular, homogeneous hypoechogenic, absent papillary growths

## Age

Age is a significant independent risk factor for ovarian malignancy in the general population, with the incidence increasing sharply after the onset of menopause. The frequency of ovarian cancer increases markedly with age, being relatively rare before age 50.^2^ The risk of malignancy is higher in postmenopausal than premenopausal women. However, most adnexal masses in postmenopausal women are benign neoplasms, such as cystadenomas. Simple cysts and hemorrhagic cysts in women of reproductive age are mostly physiologic.^7^ The simple cysts in postmenopausal women are common too, and clinically inconsequential.^7^ Appropriate tests should be carried out to exclude ovarian cancer in a postmenopausal woman who developed nonspecific symptoms within the last 12 months that suggest irritable bowel syndrome, unspecified gastric symptoms, unexplained weight loss, increased abdominal volume. This is particularly true in women over 50 years of age or those with a significant family history of ovarian, bowel, or breast cancer.^5^

## Personal and family background

Nulliparity, early menarche, late menopause, caucasian race, primary infertility, and endometriosis are contributing factors for a higher risk for ovarian cancer.^1^ Nevertheless, the most critical personal risk factor for ovarian cancer is a strong personal or family history of breast or ovarian cancer, as they may be carriers of deleterious mutations in genes related to these two types of cancer. Most gynecological cancers are sporadic, but approximately 10–18% of OC has a hereditary pattern that attributed to mutations in one of the BRCA genes.^8^ BRCA1and BRCA2 mutations confer a lifetime risk for developing OC of 39–46 % and 11–27%, respectively.^9^ Other genes besides BRCA1 and BRCA2 are also related to ovarian cancer.^10^ Until their 70 years of age, women with Lynch syndrome have a 5–10% estimated risk for ovarian cancer.^1^ When the personal or family history suggests a high risk to hereditary ovarian-breast cancer predisposition, a geneticist should be consulted.

## Symptoms and physical examination

Patients with symptomatic adnexal masses, especially climacteric, have a higher risk of malignancy.^2^ Ovarian cancer presents nonspecific symptoms within the last 12 months mimicking irritable bowel syndrome, unspecified gastric symptoms, fatigue, and unexplained weight loss. More specifically, infiltrative or compressive signs observed when increasing abdominal volume leading to pelvic pain, bowel habits modification, abnormal uterine bleeding, and a feeling of bladder fullness are noted. These symptoms appear quickly, are recent and persistent.^10^ ^11^ Although the physical examination has low sensitivity for detecting adnexal masses, it can provide some criteria for distinguishing between benign and malignant lesions (Chart 2).

**Chart 2:** Symptoms and findings on physical examination suggestive of malignancy

Symptoms	Physical examination findings
Pain (pelvic, abdominal, or back), bundling, increased abdominal volume, multiple symptoms, the persistence of symptoms	Large adnexal mass, fixed mass, irregularity, ascites

## Imaging

Transvaginal ultrasonography (TVUS) is the single most effective way of evaluating an ovarian mass.^1^ ^6^ Computed tomography (CT), magnetic resonance imaging (MRI), and positron emission tomography (PET) are not recommended in the initial evaluation of adnexal masses. The size and composition of the mass (cystic, solid, or mixed), its laterality, as well as the presence or absence of septations, mural nodules, papillary excrescences, or free fluid in the pelvis, should be assessed through TVUS. For the evaluation of vascular features of lesions in the pelvis, spectral, color Doppler ultrasound can be helpful.^1^ The morphological aspects present on TVUS that suggest malignancy are (1) irregular and thick walls and septa; (2) papillary projections; (3) solid injuries; (4) moderate echogenicity at the ultrasound.

The big ovarian and the extra-ovarian masses should be evaluated using both transvaginal and transabdominal ultrasonography approaches.^6^ The Color Doppler findings improve the morphology assessment on ovarian cancer risk instead of used alone to adnexal mass evaluation.^12^ If one hand, computed tomography (CT), magnetic resonance imaging (MRI), and positron emission tomography (PET) ) should be avoided on the first assessment of adnexal masses^1^ ^6^ in complex lesions, these new imaging approaches may be useful.^6^ If ultrasonography is inconclusive to characterize indeterminate ovarian cysts, MRI can be the second-line imaging option.^5^ ^12^ Computed tomography is the best approach for suspected extra ovarian disease or when it has to be rule out.^12^

The IOTA group standardizes criteria for the classification of adnexal masses according to characteristics of the ovarian surface, presence of septa, papillary vegetation, cyst wall, and vascularization. The IOTA group proposed two systems for estimating the risk of malignancy in adnexal masses. According to “The Ultrasound Simple Rules,” masses are classified as benign, malignant, and inconclusive, and in the “ADNEX “, is used a cutoff of 10% to predict malignancy.^13^ The systems have a sensitivity of 92% and 96.5% and specificity of 96% and 71.3%, respectively, for benign and malignant masses.^14^ We highlight that none of those instruments should use for screening for ovarian cancer, but only for referral to general hospitals or referral hospitals for treatment.^15^

## Serum tumor markers

Tumor markers can be used alone or in combination with imaging tests and clinical information for the differential diagnosis of adnexal masses. Serum marker testing indicates the likelihood of malignancy and the need for surgery.^1^

The CA125 transmembrane glycoprotein is elevated in 80% of ovarian carcinomas, especially in advanced tumors.^16^ This tumor marker is the most used to differentiate benign and malignant adnexal masses. The sensitivity rates of CA125 differentiating benign and malignant conditions ranges from 61% to 90%. The specificity rates range from 71% to 93%. The positive and negative predictive value range from 35% to 91%, and 67% to 90%, respectively.^17^ CA125 is elevated in less than half of women with initial ovarian carcinoma and may be elevated in women with benign premenopausal diseases, which include physiological conditions, endometriosis, pregnancy and menstruation.^18^ CA125 levels alone should not be used to determine the malignancy of adnexal mass. While a very high value may assist in reaching the diagnosis, an average rate does not exclude ovarian cancer due to the nonspecific nature of the test.^5^

A serum CA-125 assay does not need to be undertaken in all premenopausal women when an ultrasonographic diagnosis of a simple ovarian cyst has been made.^6^ If serum CA-125 assay more than 200 units/ml, discussion with a gynecologic oncologist is recommended.^6^

HE4 (human epididymis protein 4) is a protein involved in sperm maturation that increases in some types of ovarian malignancies and has been used in the differential diagnosis of adnexal masses.^19^ In addition to malignant neoplasms, different other factors influence serum concentrations of HE4. Variations occur with age, smoking, chronic kidney disease, but not with the menstrual cycle, contraceptives, and endometriosis, which makes this marker useful in these situations.^20^ ^21^ Lactate dehydrogenase (LDH), α-fetoprotein (α-FP) and hCG should be measured in all women under age 40 with a complex ovarian mass because of the possibility of germ cell tumors.^6^

## Multimodal tests

The effectiveness of using panels of biomarkers combined with clinical and radiologic evaluation for the distinction between benign and malignant adnexal masses has been studied.^1^ In adnexal mass surgical cases, using serum biomarker panels can be an alternative to the CA 125 level alone for assessing the need for referrals to gynecologic oncology. Although these biomarker panels should not be used in the initial evaluation of adnexal masses, they can help determine the patient that can benefit from referrals to gynecologic oncology.^1^ Currently, there is no strong enough evidence to recommend a particular test.

The risk of malignancy index (RMI) algorithm combines the value of CA 125 serum levels, ultrasound, and menopausal status. It is used to assess the risk of malignancy and calculated using the following formula RMI = U x M x CA 125 (U = score, M = menopausal status, serum levels of CA 125).^22^ When using the RMI 200 cutoff, the sensitivity and specificity of the method are 85% and 97%, respectively. Patients with values greater than 200 are at 42 times greater risk of cancer than patients with an RMI of 0.15. A systematic review of diagnostic studies concluded that the RMI I is the most effective for women with suspected ovarian cancer.^6^

The most frequent use of HE4 is for the assessment of the risk of malignancy through the ROMA (Risk of Ovarian Malignancy Algorithm) algorithm, which is a quantitative test combining the concentration of CA 125, HE4 and menopausal status.^23^ This test is calculated using two logistic regression formulas separately for peri and postmenopausal women by considering the logarithm of CA 125 and HE4 concentration.^24^ ^25^ None of these tests; CA 125, HE4 alone, RMI and ROMA have specificity to differentiate malignant from benign adnexal masses categorically. However, they are useful to assess the risk, and together with clinical and imaging information, determine whether the patient can have expectant management, investigation in general hospitals or referral to oncologic centers is recommended because of high risk for malignant neoplasm. HE4 is useful in differentiating adnexal masses with elevated CA 125 and suggestive of endometriosis, as it does not undergo major changes in the latter condition.^24^

## Management of adnexal mass

### Is the patient's age important to define the management?

The incidence of adnexal masses in childhood and adolescence is very low, higher in the first year of life due to hormonal phenomena in utero, and rises again close to menarche. The proportion of malignant neoplasms is higher in prepubertal women than in menacme.^26^ For these reasons, any adnexal mass with a solid component in this age group should be investigated with the anatomopathological examination. The therapeutic approach must include the differential diagnosis of malignancy and the hormonal and reproductive aspects of the patient. Whenever possible, a minimally invasive procedure focused on preserving the ovaries is recommended. Teratomas, the most common germ cell tumors, can and should be removed without sacrificing the rest of the ovary. Even malignant germ tumors allow conservative management.^27^

In menacme, benign adnexal masses are treated by cystectomies, oophorectomies or salpingo-oophorectomies in more than a third of cases. In patients close to menopause, this number is close to 50%. In borderline tumors, oophorectomies with or without salpingectomy are performed in about 70% of cases in this age group.^28^ However, in recent years, there has been a trend to preserve ovaries in benign ovarian masses. This approach seems appropriate because even considering that the ovaries are paired organs, preservation should always be attempted in the face of benign diseases in young women. In women close to menopause, even with ovarian preservation, opportunistic salpingectomy has been increasingly recommended because of new concepts related to ovarian carcinogenesis.^29^ High-grade serous carcinoma originates in the tubal epithelium.^30^ ^31^ ^32^ ^33^

Most ovarian carcinomas occur in women over 50 years of age. It is recommended that ovarian cysts in postmenopausal women should be initially assessed by measuring serum CA125 level and transvaginal ultrasound scan.^5^ Ovarian carcinomas should be treated in referral centers due to the high morbidity and mortality of this disease. Approximately 25% of patients with high-grade ovarian serous carcinoma die within the first ninety days, and 40% die before completing the first year of diagnosis.^34^ Patients treated in general hospitals who not adhere to strict protocols compared to referral centers have an overall survival in five years of 11.4 versus 49.5 months, respectively.^35^ The centralization of the treatment of ovarian carcinoma in referral centers has demonstrated a considerable increase in overall survival.^36^

### Why adopt conservative management?

Ovarian cancer, while typically cystic, does not arise from these benign-appearing cysts. In premenopausal women, after a good quality ultrasound in women of reproductive age, don't recommend follow-up for a classic corpus luteum or simple cyst <5 cm in greatest diameter. Use 1 cm as a threshold for simple cysts in postmenopausal women.^7^ Women with small (less than 50 mm diameter) simple ovarian cysts generally do not require follow-up as these cysts are very likely to be physiological and almost always resolve within 3 menstrual cycles. Women with simple ovarian cysts of 50–70 mm in diameter should have yearly ultrasound follow-up, and those with larger simple cysts should be considered for either further imaging (MRI) or surgical intervention.^6^ Ovarian cysts that persist or increase in size are unlikely to be functional and may warrant surgical management.^6^ Combining the oral contraceptive pill does not promote the resolution of functional ovarian cysts.^6^

In postmenopausal women, asymptomatic, simple, unilateral, unilocular ovarian cysts, less than 5 cm in diameter, have a low risk of malignancy. In the presence of normal serum CA125 levels, these cysts can be managed conservatively, with a repeat evaluation in 4–6 months. It is reasonable to discharge these women from follow-up after one year if the cyst remains unchanged or reduces in size, with normal CA125, taking into consideration the woman's wishes and surgical fitness.^5^ If a woman is symptomatic, further surgical evaluation is necessary. A woman with a suspicious or persistent complex adnexal mass needs surgical assessment.^5^

### What is a better surgical approach?

The minimally invasive surgery (MIS) is a well-established route in propaedeutic and treatment of benign adnexal masses and has been progressively indicated in oncology. This approach has significant advantages, with careful selection of patients and not to disseminate neoplastic cells.^37^ In women undergoing surgery for benign ovarian tumors, laparoscopy was associated with a reduction in fever, urinary tract infection, postoperative complications, postoperative pain, hospitalization, and total cost.^38^ Spillage of cyst contents should always be avoided, as pre and intraoperative assessment cannot absolutely preclude malignancy.^6^ The surgical specimen should be removed from abdominal cavity without intraperitoneal spillage in the plastic retrieval bag through the umbilical port, small Pfannenstiel incision, or transvaginally.^5^ The rupture alters the staging in the event of malignancy and may indicate adjuvant chemotherapy for this reason alone. Aspiration is not recommended for the management of ovarian cysts in postmenopausal women except for the purposes of symptom control in women with advanced malignancy who are unfit to undergo surgery or further intervention.^5^ In the presence of large masses with solid components (for example large dermoid cysts) laparotomy may be appropriate.^6^

### Reasons for referrals to gynecologic oncology

When a patient with a suspicious or persistent complex adnexal mass requires surgical evaluation, a physician trained to appropriately stage and debulk ovarian cancer, such as a gynecologic oncologist, should perform the operation. Below are listed the criteria (one or more should be met) of the American College of Obstetricians and Gynecologists for referring women with an adnexal mass to gynecologic oncology:^1^

- Postmenopausal, high level of CA 125, US characteristics of malignancy, ascites, nodular or fixed pelvic mass, or evidence of abdominal or distant metastasis;
- Premenopausal, high level of CA 125, US characteristics of malignancy, ascites, nodular or fixed pelvic mass, or evidence of abdominal or distant metastasis;
- Premenopausal or postmenopausal, risk assessment high score in formal tests such as the multivariate index assay, risk of malignancy index, the Risk of Ovarian Malignancy Algorithm, or one of the ultrasound-based scoring systems from the International Ovarian Tumor Analysis group.

The National Comprehensive Cancer Network (NCCN) recommends an evaluation by a gynecologic oncologist for all patients with suspected ovarian malignancies; published data demonstrate that primary assessment and debulking by gynecologic oncologist result in a survival advantage.^3^

### What is the value of the frozen section intraoperative examination?

Frozen sections for the intraoperative diagnosis of a suspicious adnexal mass is recommended in settings in which availability and patient preference allow.^12^ This recommendation is based on a meta-analysis of frozen section diagnoses that included 38 studies, involving 11,181 participants, and yielded an overall sensitivity of 90.0% (95% confidence interval (CI) 87.6% to 92.0%); with most studies typically reporting range of 71% to 100%), and average specificity was 99.5% (95% CI 99.2% to 99.7%; range 96% to 100%). If the frozen section showed a benign or invasive cancer, the final diagnosis would remain the same in, on average, 94% and 99% of cases, respectively. In cases where the frozen section diagnosis was a borderline tumor, on average 21% of the final diagnoses would turn out to be invasive cancer.^39^ In case of doubt and in order to preserve the ovary, it is reasonable to remove only the adnexal mass without rupture or spread content in the peritoneal cavity. Then, wait for the definitive paraffin exam result to define the nature of the disease and to complete surgery if necessary.

### What to do with the diagnosis of malignancy after non-cancer surgery?

Referrals to oncology specialists for additional treatment should occur when malignancy is found during laparoscopy or after histology.^5^ Stage II to IV cases with residual and unresectable disease should be evaluated for interval debulking surgery before the fourth cycle of chemotherapy. The preference is interval debulking surgery after 3 cycles of chemotherapy and it may be performed after 4 to 6 cycles, depending on the clinical judgment of the gynecologic oncologist. Postoperative chemotherapy may be advised after analysis of surgical results. All stage II-IV patients with suspected residual and potentially resectable disease should undergo tumor reduction surgery.^3^

## Final considerations

Adnexal masses are anomalies that affect women of all ages, from the earliest childhood to senility. They are more common in menacme, where the occurrence of benign diseases is also greater. At the extremes of life, in pre-adolescence and postmenopause, diagnoses of malignancy are more frequent. There are recommendations against routine screening for ovarian cancer, including use of transvaginal ultrasonography, CA 125 level, and screening pelvic examination. The differential diagnosis between benign adnexal masses is made by clinical history, ultrasound, other imaging methods and tumor markers. No method alone or in combination has sufficient sensitivity and specificity to formalize the diagnosis of malignancy. However, they are useful to differentiate patients with low probability of malignancy, who can be treated in general hospitals, from those with a high probability of malignancy, who must be treated in referral centers with multidisciplinary teams and high volume, within defined protocols. In benign adnexal masses, minimally invasive surgery should be the route of choice. The systematic removal of ovaries in benign ovarian diseases has given way to surgeries with conservation of the gonads.

**National Specialized Commission on Gynecologic Oncology of the Brazilian Federation of Gynecology and Obstetrics Associations (FEBRASGO)**

President:

Walquíria Quida Salles Pereira Primo

Members:

Angélica Nogueira Rodrigues

Caetano da Silva Cardial

Delzio Salgado Bicalho

Eduardo Batista Candido

Francisco José Cândido dos Reis

Jesus Paula Carvalho

Marcia Luiza Appel Binda

Renato Moretti Marques

Ricardo dos Reis

Sophie Françoise Mauricette Derchain

Suzana Arenhart Pessini
